# Dynamic Energy Landscapes of Riboswitches Help Interpret Conformational Rearrangements and Function

**DOI:** 10.1371/journal.pcbi.1002368

**Published:** 2012-02-16

**Authors:** Giulio Quarta, Ken Sin, Tamar Schlick

**Affiliations:** 1 Department of Chemistry, New York University, New York, New York, United States of America; 2 Howard Hughes Medical Institute - Medical Research Fellows Program, Chevy Chase, Maryland, United States of America; 3 Courant Institute of Mathematical Sciences, New York University, New York, New York, United States of America; Columbia University, United States of America

## Abstract

Riboswitches are RNAs that modulate gene expression by ligand-induced conformational changes. However, the way in which sequence dictates alternative folding pathways of gene regulation remains unclear. In this study, we compute energy landscapes, which describe the accessible secondary structures for a range of sequence lengths, to analyze the transcriptional process as a given sequence elongates to full length. In line with experimental evidence, we find that most riboswitch landscapes can be characterized by three broad classes as a function of sequence length in terms of the distribution and barrier type of the conformational clusters: low-barrier landscape with an ensemble of different conformations in equilibrium before encountering a substrate; barrier-free landscape in which a direct, dominant “downhill” pathway to the minimum free energy structure is apparent; and a barrier-dominated landscape with two isolated conformational states, each associated with a different biological function. Sharing concepts with the “new view” of protein folding energy landscapes, we term the three sequence ranges above as the sensing, downhill folding, and functional windows, respectively. We find that these energy landscape patterns are conserved in various riboswitch classes, though the order of the windows may vary. In fact, the order of the three windows suggests either kinetic or thermodynamic control of ligand binding. These findings help understand riboswitch structure/function relationships and open new avenues to riboswitch design.

## Author Summary

Riboswitches are RNAs that modulate gene expression by ligand-induced conformational changes. However, the way that sequence dictates alternative folding pathways of gene regulation remains unclear. In this study, we mimic transcription by computing energy landscapes which describe accessible secondary structures for a range of sequence lengths. Consistent with experimental evidence, we find that most riboswitch landscapes can be characterized by three broad classes as a function of sequence length in terms of the distribution and barrier type of the conformational clusters: Low-barrier landscape with an ensemble of conformations in equilibrium before encountering a substrate; barrier-free landscape with a dominant “downhill” pathway to the minimum free energy structure; and barrier-dominated landscape with two isolated conformational states with different functions. Sharing concepts with the “new view” of protein folding energy landscapes, we term the three sequence ranges above as the sensing, downhill folding, and functional windows, respectively. We find that these energy landscape patterns are conserved between riboswitch classes, though the order of the windows may vary. In fact, the order of the three windows suggests either kinetic or thermodynamic control of ligand binding. These findings help understand riboswitch structure/function relationships and open new avenues to riboswitch design.

## Introduction

Riboswitches are RNAs in the untranslated (UTR) regions of messenger RNAs (mRNAs) that can undergo a structural transition in response to a highly specific intracellular ligand [1]–[4]. Once bound to the riboswitch, the ligand induces a rearrangement on the secondary structure level. The new conformation can turn on or off transcription [5]–[7] or translation [8]–[11]. An additional mechanism for gene control has been recently discovered in which eukaryotic riboswitches control sequestration or opening of key alternative mRNA splice sites [12], [13]. Currently, more than twenty classes of riboswitches are known and classified according to their cognate intracellular metabolite [4]. This list of ligands that bind riboswitches has expanded from small molecule metabolites to include second messengers such as cyclic di-guanosine monophosphate (cdGMP) [14]–[16], other RNAs [17], and possibly hormones [18].

**Figure 1:**
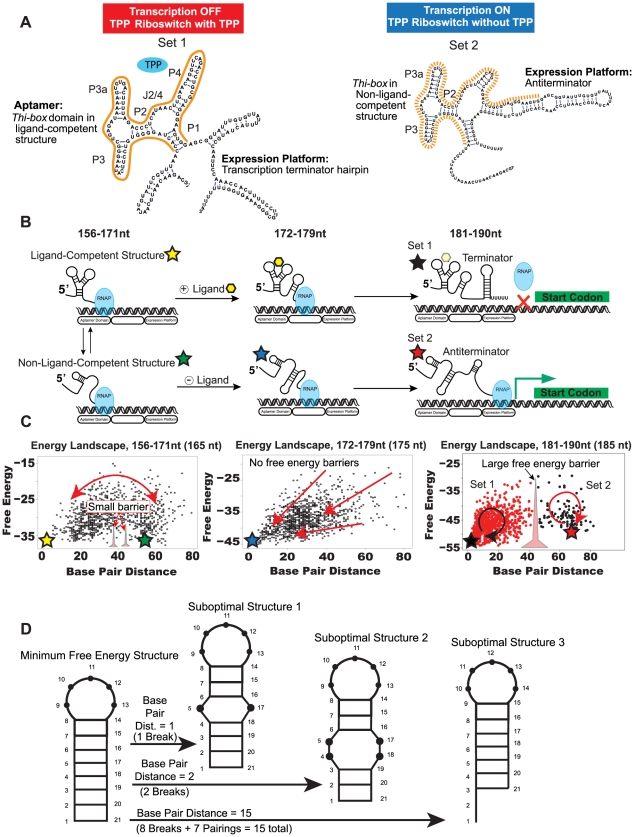
The riboswitch control of gene regulation. (a) The two full length structures of the *tenA* thiamine pyrophosphate (TPP, blue oval) riboswitch are shown. Aptamer domain is highlighted in orange solid and broken lines. (b) Simplified diagram of riboswitch folding process for the *tenA* TPP riboswitch. From 156–171 nt, meta-stable structures (labeled ligand-competent and non-ligand-competent) exchange with one another. From 172–179 nt, ligand (yellow polygon) stabilizes one of the ligand-competent meta-stable structures, thus causing the formation of specific terminator hairpin structures in the full length riboswitch (181–190 nt). The ligand may remain bound or disengage the aptamer later in transcription (polygon in dashed lines in top row, right column). In the absence of ligand (172–179 nt), isomerization to a different structure occurs, causing an antiterminator structure to form downstream (181–190 nt). (c) The energy landscapes of the riboswitch through all three stages of transcription. Each point represents a different secondary structure; marked according to its base pair distance (structure distance) and free energy. Colored stars represent points on the landscape corresponding to structures in (1b). In the energy landscape from 156–171 nt, represented here by the landscape at 165 nt, a small energy barrier and multiple low-energy structures exist on the landscape simultaneously, permitting exchange of meta-stable structures (double arrow). From 172–179 nt (shown for 175 nt), the landscapes are funnel-shaped and cause isomerization to the mfe if ligand is not present to stabilize the ligand-competent structure. From 181–190 nt (shown here for 185 nt), two structures are possible which the RNA may fold into. The high energy barrier between sets precludes switching. Set 1 corresponds to terminator structures and Set 2 corresponds to antiterminator structures. (d) Example calculation of base-pair distance for a simple helix structure.

Riboswitches are composed of two major RNA domains: an aptamer domain, which binds the ligand, and an expression platform, which controls gene expression (Figure 1a). The aptamer is the first portion of the riboswitch's sequence and is defined by its ability to fold into a higher-ordered structure that can bind the ligand. As the aptamer is transcribed, fast base-pairings occur, forming a specific structure to which the ligand may bind called the “ligand-competent” or “pre-organized” state (Figure 1b, first row). However, non-ligand-competent structures are also possible (Figure 1b, second row). In the event that the ligand-competent structure docks its target ligand, a specific structure in the downstream expression platform forms (Figure 1a, b).

One of the most common forms of gene control by the expression platform is the transcription terminator hairpin. As illustrated in Figure 1, the binding of thiamine pyrophosphate (TPP) to the ligand-competent structure of the aptamer domain forces a transcription terminator hairpin to form in the expression platform, which inhibits RNA polymerase from proceeding. If TPP does not bind to the aptamer structure, the expression platform forms a different structure, termed antiterminator, which allows RNA polymerase to transcribe the downstream gene (Figure 1a, right; Figure 1b, bottom row).

Another example of gene control by the expression platform is the folding of the Shine-Dalgarno sequence. The Shine-Dalgarno sequence is a section of the expression platform and the ribosome binding site in prokaryotes. In the presence of ligand, the Shine-Dalgarno sequence forms a double-stranded RNA (anti-SD), which prevents the ribosome from binding and precludes translation of the gene. Transcription termination and Shine-Dalgarno sequence sequestration are both mechanisms that riboswitches use to control gene expression; however, they accomplish this by acting on two different processes within the cell [19].

To understand riboswitch gene control *in vivo*, the RNA folding process must be investigated. RNAs begin to fold as they are transcribed in the cell and are efficiently directed toward a stable conformation through fast base-pairing interactions (∼100 ms) [20], [21]. Thus, meta-stable folded structures of the available sequence fraction are thought to form quickly and differ from the native states of the full length RNA (Figure 1b). A meta-stable folded structure is any combination of base-pairings for a shorter-than-full length RNA sequence. RNA elongation also fluctuates due to pause sites and variations in polymerase speed, affecting the fraction of sequence available for folding [22]. Meta-stable intermediates may not rearrange to the full length native conformation, because dissociation of structural elements might be energetically costly, resulting in a kinetic stabilization (“trapping”). All these intrinsic properties of transcription affect RNA folding *in vivo* [23]–[26].

Recently, studies have elucidated two mechanisms of ligand binding in riboswitches: thermodynamic and kinetic [27]. The mechanism of ligand binding involves a two-step chemical reaction, as follows.
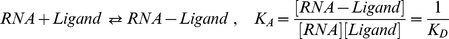
As with any reaction proceeding toward equilibrium, time is needed for reactants to be consumed and for products to be formed. However, the process of *in vivo* folding places limits on the time permitted for RNA-ligand equilibration. First, in the absence of transcriptional pause sites, RNA polymerase transcribes nucleotides quickly; ligand binding occurs before the polymerase reaches the end of the expression platform (Figure 1b, right side). If the ligand cannot bind in time, proper folding of the RNA will not occur, and gene regulation cannot occur. The second limitation to RNA-ligand equilibrium is formation of meta-stable intermediates, which hamper or eliminate ligand binding to the aptamer domain by altering the structure of the ligand binding pocket (Figure 1b, bottom). Work has shown that high concentrations of ligand are required for gene regulation to occur *in vivo* and that these concentrations surpass the *in vitro* dissociation constant (K_D_) [22], [28]–[30]. This setting is the hallmark of kinetic control of ligand binding [22]. Kinetic control primarily relies on the rate of ligand binding and RNA transcription. A high ligand concentration drives the above mentioned equilibrium toward the RNA-ligand complex. In contrast, thermodynamic control occurs when the ligand greatly stabilizes the RNA and reaches equilibrium in a time frame shorter than the time of transcription. In this case, the K_D_ of the aptamer-ligand complex is generally near the cellular concentration of the ligand [30].

A riboswitch may use both strategies, as shown for the *pbuE* riboswitch [30]. When more time is permitted for transcription, as through use of transcription pausing, the riboswitch can reach equilibrium with ligand. However, when transcription time is shortened, greater concentrations of ligand are required for gene regulation to occur, and the riboswitch operates under kinetic control. Presumably because of differences in the mechanism of gene control, ligand binding affinities vary widely among riboswitch classes (Table 1). These variations are related to the concentration of ligand needed to elicit gene control *in vivo*. For example, although both *pbuE* and *add* riboswitches bind adenine, *pbuE* demonstrates kinetic control, while *add* shows thermodynamic control [31].

**Table 1:** Riboswitch classes in present study.

Riboswitch Class	Wild-Type Analyzed	Dissociation Constant (K_D_) *	Concentration sufficient for structural change	Mechanism	Ref
Thiamine Pyrophosphate (TPP)	1. *tenA Bacillus Subtilis* (transcription)	50–500 nM	50–100 µM	Kinetic	[19], [68], [69]
Thiamine Pyrophosphate (TPP)	2. *thiM Escherichia coli* (translation)	50–500 nM	50–100 µM	Kinetic	[19], [68], [69]
Molybdenum cofactor (Moco)	3. *moaA Escherichia coli* (translation)	Unknown	10 mM	*Kinetic (proposed here)*	[70]
Guanine	4. *xpt Bacillus subtilis* (transcription)	5–50 nM	1–10 µM	Kinetic	[108]
Adenine	5. *pbuE* (*ydhL*) *Bacillus subtilis* (transcription)	300–574 nM	500 µM	Kinetic or Thermodynamic‡	[30], [31], [76], [81]
Adenine	6. *add* Adenine *Vibrio vulnificus* (translation)	440–680 nM	2340 nM	Thermodynamic	[31]
Magnesium (Mg)	7. *mgtE Bacillus subtilis* (transcription)	Unknown	2–10 mM	*Kinetic (proposed here)*	[74], [109]
Cyclic-di-Guanosine Monophosphate (cdGMP, GEMM)	8. *Candidatus Desulforudis audaxviator* (transcription)	1 nM	100 µM	Kinetic	[15], [16]
Pre-queosine (PreQ1, PreQI-II)§	9. *Fusobacterium nucleatum* (transcription)	200–300 nM	1 µM	Kinetic or Thermodyanmic‡	[64]
S-adenosylmethionine (SAM)§	10. *metI Bacillus subtilis* (transcription)	4–20 nM	1–50 µM	Kinetic	[5], [73], [110]

Thermodynamic and kinetic control of ligand binding is defined in the Introduction. Briefly, if the time required for equilibrium between ligand and aptamer is equal to or less than the amount of time it takes for the RNA to be fully transcribed, the switch will approximate thermodynamic (equilibrium) control. However, if the time required for RNA-ligand equilibrium is long, the switch is under kinetic control.

*K_D_ values listed are for the aptamer sequence at 298K, but ranges depend on experimental conditions.

**‡:** Investigators proposed that these riboswitches could function under thermodynamic control if there were transcriptional pause sites of a couple seconds or changes from standard temperature.

**§:** These riboswitches are known to require pseudoknot interactions for activity.

Riboswitch folding is a multi-step hierarchal process, involving interactions between base-pairs (Watson-Crick A-U, G-C, and G-U wobble), base stacking, hydrogen bonding, and tertiary interactions between distant or proximal nucleotides. While gene control is affected by changing secondary structure, local changes also occur to adopt a binding pocket specific for a small ligand. Modeling RNA interactions on the global and local levels is thus required to fully grasp the switching process (for a review of RNA modeling see [32], [33]).

RNA secondary structure can be predicted from a single sequence or multiple aligned sequences to produce the base pairing arrangement that yields the minimum free energy structure as well as nearby low-energy states. Algorithms may use thermodynamic models to predict structures with low Gibbs free energy [34], use prior knowledge of validated structures to predict probable structures [35], [36], or search for a structure common to multiple sequences [37]–[40]. However, predicting 2D structures is limited by thermodynamic parameters, which are subject to inaccuracies measured experimentally and simplified functional forms used. Sampling multiple, suboptimal structures provides a more global view that addresses in part parameter uncertainties.

In addition to the platform provided by secondary structure, tertiary contacts further stabilize specific conformations. Programs developed over recent years take different approaches to the problem of RNA folding; see recent perspectives [32], [33]. One of the first programs to accurately predict the structure of RNA was FARNA, an energy-based program that simplifies each base as a single bead representation. The program uses prior knowledge of solved rRNA structures and secondary structure input to predict the conformation of the RNA being analyzed. Using this method, FARNA reached an average RMSD∼30 Å in predicting the structure of the *Tetrahymena ribozyme* [41].

Another interesting approach, used by the programs MC-Sym and NAST, involves the input of secondary and tertiary structure constraints to produce 3D RNA structures. The MC-Fold and MC-Sym pipeline use both base pairing and base stacking interactions to build sets of nucleotide cyclic motifs that define RNA structure [42]. Using experimental data on the tertiary contacts of the HDV ribozyme, Reymond et. al. used MC-Sym to map out individual folding intermediates [43]. NAST was recently developed to employ molecular dynamics sampling of a coarse-grained model based on knowledge-based statistical potentials [44]. For example, with some tertiary contact information, compact states of the *Tetrahymena ribozyme* could be predicted [45]. A comparative evaluation of some of these approaches has been made in [33], and a recent review [32] also discusses many limitations.

Previous modeling studies have explored two aspects of aptamer folding: folding in the presence of ligand, and self-directed folding (without ligand). It is believed that most of the structural scaffolding, which includes secondary and tertiary interactions, is quickly formed, while the addition of the ligand only causes specific tertiary contacts. For example, Stoddard et. al. [46] revealed that an ensemble of ligand-competent conformations occurs for the SAM aptamer, distinguished only by large-scale relative motion of helices. Therefore, SAM captures a ligand-competent conformation with most of the structure pre-organized, and this is followed by local adjustments to reach the fully “native” state. In addition, dynamics simulations have revealed that in the process of SAM binding, a core portion of the aptamer region is stabilized significantly, indicating that the majority of the binding pocket is pre-formed [47]. Furthermore, Villa and colleagues [48] found a two-step process in the guanine sensing aptamer: A primary screening step for purine molecules is followed by highly discriminative selection for guanine, suggesting that the pocket forms in the absence of guanine. In the related adenine riboswitch, Sharma et. al. [49] show a similar stepwise mechanism for ligand binding.

In cooperation with the pre-organized aptamer, key tertiary interactions form when the ligand binds, and prior simulations have also shown how this response to the ligand occurs. An atomic-level computer simulation of the S-adenosylmethionine (SAM) aptamer [50] showed that the fully folded structure is formed only after binding of the ligand, which reduces the barrier to folding and triggers helix formation. In support of these computational results, Wilson et. al. have shown by NMR that certain conformations form exclusively in the presence of SAM [51]. Similar results have been obtained for multiple aptamer classes including the preQ_1_ [52] and adenine aptamers [53]. In addition, SAM stabilizes a key subset of tertiary interactions distant from the binding pocket, functioning to collapse the aptamer and control secondary structure switching [54].

To better interpret the folding process of RNA, we use the perspective of the “new view” of protein folding, which relies on the concept of a free energy landscape [55]. The free energy landscape is defined by the ensemble free energies of all conformations where each conformation is associated with an energy and distance measure with respect to all other conformations [56] (Figure 1c) as evaluated by our computational approach (see Materials and Methods). Here, we use the base pair distance as a generalization of distance measure between RNA conformations, akin to the root mean square distance (RMSD) in protein structure (Figure 1d). This base pair distance is essentially the difference in Watson-Crick base-pairs between two structures [57], [58].

In general, biological molecules take advantage of a funnel-shaped landscape representing many high-energy (denatured) conformations and few low-energy states. This arrangement permits the sequence to search the astronomical number of conformations directly and efficiently. In a “smooth” energy landscape, there are few low-energy structures in the lowest energy portion of the funnel, whereas a “rough” energy landscape has more low-energy structures with barriers between them. In the latter, each of the low-energy structures has a smaller funnel leading to it. If the landscape is smooth and has a single minimum, the minimum free energy occurs near the native state. This situation is called “downhill folding [59].” In downhill folding, there is little or no free-energy barrier, and folding occurs quickly (Figure 1c, middle). In contrast, “barrier-limited folding” landscapes are “rougher” or “frustrated” and are marked by the presence of one or more low-energy barriers, which slow transition times and affect pathways to the minimum energy structure (Figure 1c, left) [59]. Feng et. al. previously demonstrated this type of energy landscape for the preQ1 riboswitch, in which stability of individual structures was linked to the rate of folding [52]. For proteins, an energy landscape is typically computed at the full sequence length. Here, we compute many landscapes at 1 nt increments to mimic folding as the sequence is transcribed. We then group similar landscapes into one landscape that captures behavior at that sequence range. For the entire elongation process, we have distinguished at most three different windows or landscapes of behavior.

We use this procedure to analyze ten riboswitches from seven different classes, by the technique we developed in [57] for the *tenA* TPP riboswitch. The nature of the unbound state, the change in secondary structure, and the effects of the expression platform on folding are all questions we address here by deriving a novel energy landscape model and validating our predictions with experimental measurements. Studies on the full riboswitch, aptamer and expression platform, are still lacking. Here, we simulate *in vivo* formation of structures by calculating the energy landscape of secondary structures sequentially from short to full length sequence, without any ligand, at 1 nt increments. Prediction of individual RNA secondary structures at different lengths is performed with a set of programs from the Vienna RNA folding package [60] as well as pknotsRG [61] for pseudoknot-containing riboswitches. These programs essentially predict structures on the basis of a set of nearest-neighbor approximations, assigned to the various motifs in RNA structures [34], [62], as described above. While secondary structure predictions do not account for all interactions, these predictions approximate the general structural scaffold and serve as a first-level approximation. As described above, most of the architecture is thought to be formed in the absence of ligand. Thus, our 2D energy landscapes provide an approximate picture of the available folding states accessible to the riboswitch during elongation. This folding as the sequence elongates to full length has not been examined computationally as far as we are aware.

Our analysis reveals that three main types of landscapes exist depending on the sequence length transcribed. The *sensing window* encompasses the lengths at which the riboswitch adapts to different structures, including the ligand-competent form. Overall, the ligand-competent and non-ligand-competent structures are inherent to the energy landscape (Figure 1b,c, left panels). At this length range these two states can interchange, regardless of the presence of ligand. Ligand binding induces folding toward the active conformation by shifting the equilibrium.

At other specific sequence lengths, the energy landscape displays a *downhill folding window*, which favors a low-energy structure with a specific function on gene control (Figure 1b,c middle panels). This sequence range essentially determines whether the riboswitch will turn the gene on or off.

Finally, at yet another stage of transcription, two alternative pathways are present on the landscape as two separate clusters (Figure 1b, c right panels). We term this portion of transcription the *functional window*. These energy landscapes demonstrate an irreversible decision point: Once one cluster is accessed, switching between states is not likely to occur.

By extending the landscape analysis in [57] for the TPP riboswitch to many other riboswitches, we find that although the overall features are similar, the order of these energy landscape windows varies and can suggest whether the ligand binding mechanism is governed by kinetic or thermodynamic control. That is, when the sensing window occurs early during the transcription process, as for the *tenA* riboswitch, landscape analysis suggests kinetic control; when the sensing window occurs at the end of the expression platform, as for the *add* riboswitch, thermodynamic control reigns. These energy landscape views thus help interpret riboswitch action by connecting structure to function. Implications to riboswitch design naturally arise.

## Results

### Overview

Our ten riboswitch examples in seven families consist of six from the Rfam database [63] plus the recently discovered cyclic-di-guanosine monophosphate riboswitch family [64], [65] (Table 1). We expand on our earlier computational approach [57] because two classes of riboswitches (PreQ, SAM) fold via pseudoknots (intertwined base-pair interactions). These classes require further analysis with pknotsRG [61] (Materials and Methods), which predicts pseudoknot formation as well as pseudoknot-containing suboptimal structures. We exclude riboswitch classes longer than 240 nt, since the accuracy of RNA folding markedly decreases at such lengths and the number of suboptimal foldings concomitantly increases exponentially.

In all riboswitches studied (Table 1), we found that three broad sequence length ranges displayed similar energy landscapes patterns. We term the three sequence ranges as the sensing, downhill folding, and functional windows, respectively. We found that the order of the windows predicts the mechanism of ligand binding (Table 1). The sensing window refers to the state at which the RNA is intrinsically able to sense or detect the presence of ligand. For all the sequence lengths within the sensing window, the energy landscape demonstrates that ligand-competent forms are separated from functionally opposing, non-ligand-competent structures by a small energy barrier, which creates a pathway between the two states (Figure 1c, left). These landscapes mimic a barrier-limited folding description. In contrast, the downhill folding window favors a single minimum free energy structure (mfe). Low barriers and a funnel-shape toward the minimum facilitate an efficient isomerization to the mfe (Figure 1c, middle). Lastly, the functional window displays compact clusters of structures, a high (>10 kcal/mol) energy barrier, and two opposing states (Figure 1c, right). In the following sections, we analyze our riboswitches according to the order of windows. We find that the main determinant of kinetic or thermodynamic control is whether the sensing window occurs early or late in transcription. However, both kinetically and thermodynamically-controlled riboswitches can vary the order of downhill folding and functional windows.

### Kinetically-controlled riboswitches that follow the order Sensing, Downhill Folding, Functional Window

**Figure 2:**
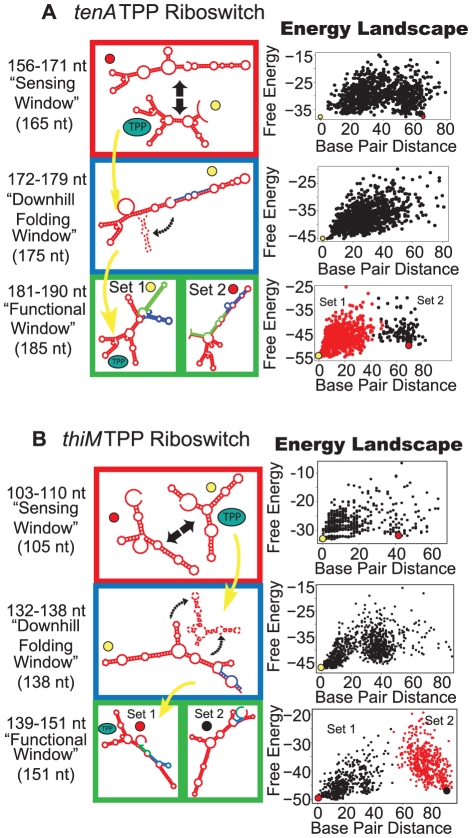
Proposed folding pathway for the TPP riboswitches *tenA* (a) and *thiM* (b). Structures formed in the sensing windows are represented in red boxes; downhill folding window structures are found in blue boxes; and functional window structures are represented inside the green boxes. Double-head arrows represent structures that can interchange. Broken-line structural elements in downhill folding window (blue box) represent structural elements that would be coerced to form in the presence of ligand. Colored circles adjacent to structures are marked by their points on the respective energy landscape to the right. Yellow arrows represent the series of structures accessed in the presence of ligand. For all sequence lengths inside of a window, the energy landscape repeatedly displays similar patterns (see Materials and Methods). The specific sequence length corresponding to the window shown is given following the length range. For full description of energy landscape characteristics see Figure S5.

Figures 2–4 show resulting landscapes for the *tenA* riboswitch from *Bacillus subtilis*, *thiM* riboswitch from *Escherichia coli*, GEMM riboswitch from *Candidatus Desulforudis audaxviator*, *moaA* riboswitch from *Escherichia coli*, and *metI* riboswitch from *Bacillus subtilis*. All riboswitches undergo conformational changes by binding specific ligands (Table 1). At the beginning of transcription in the sensing window, the ligand-competent aptamer is the mfe but non-ligand-competent structures are also present on the landscape. In the downhill folding window, an immediate change occurs as the mfe switches to the non-ligand-competent antiterminator (*tenA*, GEMM, *metI*) or anti-SD (*thiM*, *moaA*). In this time frame, the energy landscapes describe a spontaneous isomerization to the stable antiterminator/anti-SD form. We propose that this window decides the ultimate fate of the riboswitch: If the RNA is ligand-bound, it does not isomerize to antiterminator/anti-SD form, and without ligand, the RNA forms the thermodynamically-favored antiterminator/anti-SD form. In the functional window, the final set of nucleotides of the expression platform form the terminator hairpin/antiterminator or sequester/open ribosome binding site, which are energetically favored.

**Figure 3 pcbi-1002368-g003:**
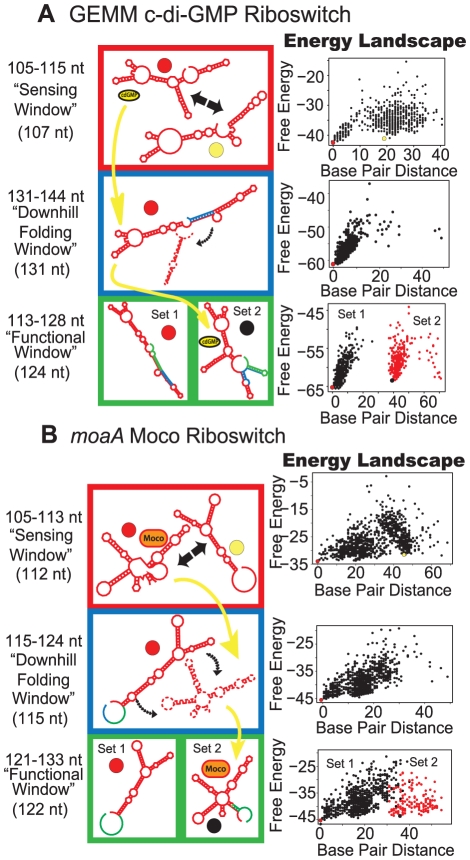
Proposed folding pathways for the GEMM (a) and *moaA* (b) riboswitches. See figure 2 caption for description of figure elements. For full description of energy landscape characteristics see Figure S5.

**Figure 4:**
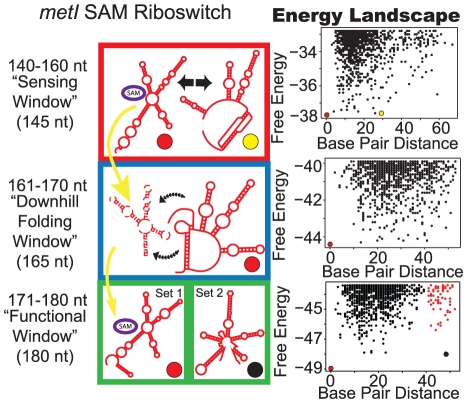
Proposed folding pathway for the S-adenosylmethionine (SAM) *metI* riboswitch. See figure 2 caption for description of figure elements. For full description of energy landscape characteristics see Figure S5.

For the *tenA* and *thiM* thiamine pyrophosphate (TPP) riboswitches, each energy landscape window correlates with several interesting experimental properties. First, the RNA favors the ligand-competent form in the sensing window (Figure 2 a,b, top). In good agreement with our computational results, pre-organization into a ligand-competent form occurs *in vitro* in the presence of relevant Mg^+2^ concentrations [66] and binds TPP with high affinity [8], [66] (Figure S1).

During the downhill folding window, the TPP riboswitch favors an antiterminator/anti-SD structure, which results in aptamer misfolding (Figure 2a,b, middle panel). Lang et al. [8] note that shorter-than-full length *thiM* riboswitch constructs, precisely at those lengths that occupied the downhill folding window, displayed hampered TPP binding. The authors conclude that alternative folds prevent TPP binding by obliterating the ligand-competent forms. Our view supports this behavior by relating the poor TPP affinity to formation of non-ligand-competent anti-SD structures.

In the full length riboswitch, experiments have shown that both *tenA* and *thiM* recognize TPP with the same affinity as the aptamer domain alone [8], [66], [67]. We also find that in the functional window, the full length TPP riboswitch favors a fully formed aptamer domain (Figure 2a,b, bottom) and has less competition from alternative folds. This stability is due to the high energy-barrier and clustering exhibited in the energy landscape. Structures distant in the *thiM* functional window (Set 2 in Figure 2b) correspond to the non-ligand-bound TPP riboswitch found experimentally. Thus, as reported by Rentmeister and colleagues [10], in the TPP-free form of *thiM*, stems P2 and P3 form, the Shine-Dalgarno sequence is unpaired, and P1 is mispaired (Figure S1).

Overall, this order of windows is characteristic of kinetic control, where the choice of folding pathway occurs early in transcription. High concentrations of ligand both stabilize the ligand-competent aptamer soon after it is transcribed and exclude non-ligand-competent forms [19]. The concentration at which transcription termination occurs [68] is much greater than the apparent K_D_ (∼50 nM) [69]. The hallmark of kinetic control is that the concentration of ligand required for *in vivo* gene regulation is greater than the binding affinity found *in vitro* (K_D_).

The GEMM riboswitch from *Candidatus Desulforudis audaxviator* belongs to a novel class of riboswitches found to bind the second messenger cyclic di-guanosine monophosphate [15], [16]. Similar to *tenA* and *thiM*, the sensing window contains ligand-competent and non-ligand-competent structures together on the energy landscape, separated by a small energy barrier (Figure 3a). Only minor differences between our predicted ligand-competent structures and the known structure can be noted (Figure S1). In the downhill folding window, the non-ligand-competent, antiterminator structure is the mfe, suggesting that the antiterminator would form if the ligand is not present to stabilize the ligand-competent structure. Similar to *tenA*, terminator and antiterminator form in the functional window. The window pattern suggests kinetic control, in agreement with experimental evidence by Sudarsan et al. [15], [16].

The molybdenum-cofactor binding *moaA* riboswitch [70] which follows the same order (Figure 3b), can bind either Molybdenum-cofactor (Moco) or Tungsten-cofactor (Tuco). Akin to *thiM*, *moaA* causes suppression of translation through sequestration of the ribosome binding site (anti-SD). The folding pathway starts with a sensing window, where ligand-competent and non-ligand-competent structures are in equilibrium. The downhill folding window that follows shows a tendency to isomerize to the anti-SD structure. Finally, in the functional window, the anti-SD forms a separate cluster from functionally-opposing structures, which have open Shine-Dalgarno sequences. This functional window does not display a clear separation of clusters as in *thiM*, though it has a high energy barrier between sets of conformations (∼12 kcal/mol). Experimental studies on kinetic or thermodynamic control of ligand binding are not yet available, though our predicted structures contain all conserved features of the ligand-bound structure (Figure S1).

The *metI* leader from *Bacillus subtilis* binds S-adenosylmethionine (SAM) and exhibits dramatic gene silencing in the presence of ligand (∼12%→75% termination in presence of ligand) [71]. The S-box aptamer requires a pseudoknot interaction for proper folding. A meta-stable, non-ligand-competent pseudoknot structure forms alongside the SAM-competent structure in our sensing window (Figure 4). However, in the downhill folding window, this non-ligand-competent structure is highly stable as the mfe, while the SAM-competent structures are unfavorable and not present in the energy landscape. In the functional window, the energy landscape demonstrates two structures, corresponding precisely to those predicted by Breaker et al. [72]. When fully formed, the terminator or antiterminator structure is essentially irreversible, as evident by high energy barriers between structures. In agreement with the irreversible structures of the functional window, Hennelly et. al. have shown that the full length SAM I antiterminator is essentially irreversible by ligand alone without refolding [54]. Both experimental [73] and computational [47] results agree with the pattern of landscape window suggesting kinetic control, because the sensing window occurs early in transcription.

### Kinetically-controlled magnesium-sensing riboswitch following the order: Downhill Folding, Sensing, Functional Window

**Figure 5:**
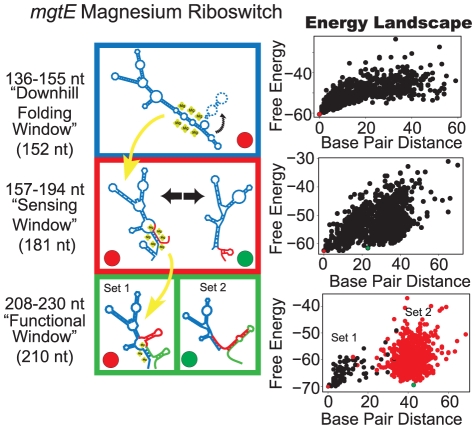
Proposed folding pathway for the *mgtE* riboswitch. See figure 2 caption for description of figure elements. For full description of energy landscape characteristics see Figure S5.

The *mgtE* riboswitch from *Bacillus subtilis* [74] is a longer RNA characterized by the presence of a terminator hairpin adjacent to the aptamer domain. As the longest riboswitch studied (230 nt), the purpose of the early downhill folding window (Figure 5) likely serves to quickly fold the long sequence into a compact, ligand-competent structure. Later in the sensing window, the ligand-competent structure exchanges with the non-ligand-competent structure. In the functional window that follows, the validated terminator and antiterminator structures exist [75] (Figure 5, S1). Although the order of windows differs from the five riboswitches above, we also propose a mechanism of kinetic control for *mgtE* ligand binding because the sensing window occurs during sequence-lengths shorter than full length. No kinetic studies have yet been performed on this riboswitch to the best of our knowledge.

### Kinetically-controlled Purine Riboswitches *pbuE* and *xpt* follow the order: Sensing, Functional, Downhill Folding Window

**Figure 6:**
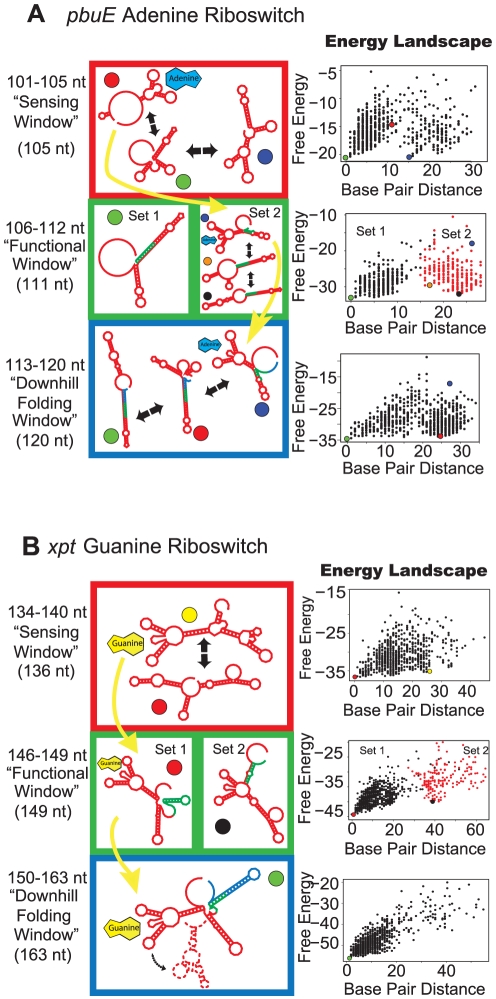
Proposed folding pathway for the *pbuE* (a) and *xpt* (b) purine riboswitches. See figure 2 caption for description of figure elements. For full description of energy landscape characteristics see Figure S5.

The *pbuE* riboswitch alters its structure in response to adenine only at short lengths [31], [76]. In agreement with NMR investigations, we predict that *pbuE* favors an adenine-binding-competent fold at short lengths, in the sensing window, where loops L2 and L3 and stem P1 forms [77] (Figure S1). However, the adenine-competent folds are higher in energy and thus buried within the clusters (Figure 6a). As a result, the *pbuE* riboswitch differs from all other classes, because the ligand-competent structure is not the mfe at any point in the windows. In strong agreement with optical trapping assays of the *pbuE* aptamer domain [78], we find that the RNA in the sensing window is in rapid equilibrium between unfolded and P1-folded (i.e., ligand-competent) states.

The sensing window in *pbuE* is followed by a functional window, in which two pathways become possible. Both Set 1 and 2 structures favor terminator hairpins (i.e., non-ligand-competent structures) (Figure 6a). Ligand-competent, antiterminator forms are buried in Set 2, and are in equilibrium with terminator structures, while Set 1 consists of non-ligand-competent structures. Thus, Set 2 represents the possible pathway in the presence of ligand, while Set 1 represents the pathway in its absence. The mfe structure of Set 2 in the functional window corresponds to a form that binds and is cleaved by RNAse P [79]. We suggest that adenine binding may signal or trigger the RNAse P interaction, since the two structures occur in the same cluster within the functional window.

The energy landscape for the full length *pbuE* RNA highly favors non-ligand-bound states as indicated by a downhill folding window toward the non-ligand-competent mfe. Adenine-competent structures exist on the landscape, but are much higher in energy. This suggests that the ligand must stabilize the RNA to prevent isomerization to more energetically favorable non-ligand-competent structures. This behavior agrees with experimental studies [76]. The full length *pbuE* riboswitch is not responsive to ligand, meaning that the RNA does not fold into a ligand-competent structure when adenine is subsequently added to solution.

Since the sensing window occurs early in transcription, *pbuE* suggests kinetic control. This finding is also in agreement with experimental results [31], although some investigators suggest that thermodynamic control may be possible through use of transcriptional pause sites and variations in temperature [30].

While the *xpt-pbuX* guanine-sensing riboswitch has a similar structure and sequence to *pbuE*, specific nucleotides in its aptamer domain bind guanine. Once the *xpt* aptamer domain is transcribed, it forms the ligand-competent structure (Figure 6b, S1) [28], [80]. Association kinetics experiments reveal that high ligand concentrations induce a unimolecular step prior to ligand binding [28], this suggests that the RNA interconverts between two isomers until the ligand-competent structure is stabilized. This result agrees with the sensing window of the *xpt* aptamer domain (Figure 6b); the mfe is ligand-competent and coexists with alternative low-energy non-ligand-competent structures on the landscape, separated by a small energy barrier.

The functional window directly follows the sensing window with the functionally-opposing terminator and antiterminator structures forming in separate clusters. The terminator structure is ligand-competent and the antiterminator structure favors breakage of the crucial P1 stem, forming a non-ligand-competent structure. Later, at the start of the downhill folding window, the mfe favors a ligand-competent, terminator form (Figure 6b). The downhill folding window at full length transcription supports isomerization to this structure, regardless of whether guanine is bound or not. However, as we argue below, isomerization is not likely to occur because of the excessive time required.

For gene regulation to occur, we propose a model of kinetic control. The structures in the sensing window likely exchange at equilibrium, much like in *pbuE*. We propose that the structure formed in the functional window is stable through the downhill folding window. Isomerization to the ligand-competent terminator mfe does not occur in the time allotted for 13 nt of the downhill folding window to be transcribed. The RNA polymerase likely transcribes without pausing, forbidding the riboswitch enough time for isomerization. If ample time (e.g., transcriptional pausing) were given to the riboswitch to fold into the preferred structure in the downhill folding window, the terminator would always form and gene regulation would not depend on the presence or absence of guanine. Whichever structures are formed at the end of the functional window (ligand-competent/terminator or non-ligand-competent/antiterminator) are thus kinetically trapped through the downhill folding window. Gene regulation can thus follow.

### Thermodynamically-controlled *add* riboswitch demonstrates a different energy landscape pattern

**Figure 7:**
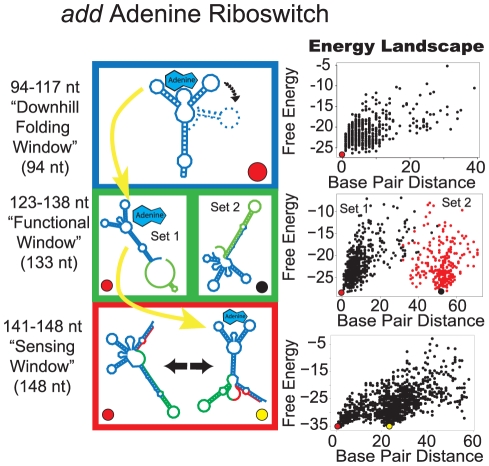
Proposed folding pathway for the *add* (a) riboswitch. See figure 2 caption for description of figure elements. For full description of energy landscape characteristics see Figure S5.

The purine aptamer domain of *add* selectively binds adenine [81] through a Watson-Crick pairing mechanism between the purine ligand and aptamer. Similar to the structures formed in the TPP riboswitches, the ligand-competent form is favored early in transcription and allows adenine to bind if it is present at adequate concentrations (Figure S1). The functional window arises immediately thereafter, isolating the available folding states. In the final stretch of transcription, the energy landscape changes into a sensing window with multiple local minima. Fluorescence experiments with 2-aminopurine (2-AP) substitution have shown that the full length riboswitch is indeed capable of binding adenine and undergoing ligand-dependent conformational changes [31]. Investigators conclude that the full length *add* riboswitch operates on the basis of thermodynamic control of adenine. Therefore, we propose that the following sequence of energy landscape windows is the hallmark of thermodynamic control (Figure 7): downhill folding window→functional window→sensing window.

### Kinetic or thermodynamic control in the preQ1 riboswitch

The preQ1 riboswitch is the smallest riboswitch class known and requires a pseudoknot interaction in the aptamer domain for ligand binding [65], [82] (Figure S1). The ligand-responsive window for the preQ1 riboswitch from *Fusobacterium nucleatum* occurs from position 35–54 relative to the transcription start site, while the full length construct still remains responsive to ligand as well [64]. Though an allosteric rearrangement from terminator to antiterminator structure is possible, preQ1 binding is significantly slower for the full length riboswitch (60.2±10.4×10^4^ M^−1^ s^−1^ vs. 7.62±0.29×10^4^ M^−1^ s^−1^ for aptamer domain and full construct, respectively). The authors propose that the riboswitch operates by kinetic control. Pausing, however, may provide ample time for the full length riboswitch to bind the ligand, alter its structure, thereby allowing thermodynamic control.

**Figure 8:**
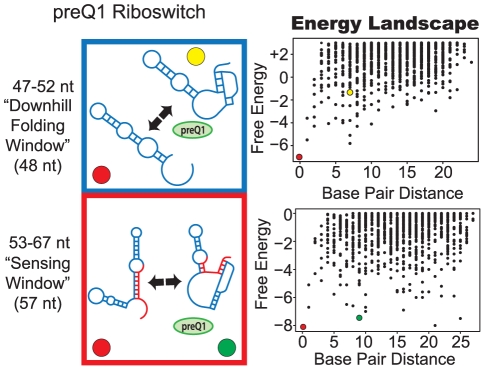
Proposed folding pathway for the preQ1 riboswitch. See figure 2 caption for description of figure elements. For full description of energy landscape characteristics see Figure S5.

Our results show that a downhill folding window occurs from positions 42 to 53, where the mfe is a non-ligand-competent structure (Figure 8). Higher-energy structures are ligand-competent; thus, the ligand-competent and non-ligand-competent structures are present on the landscape simultaneously and can interchange. At 54 nt, the expression platform begins to be transcribed, and the number of low-energy forms increases due to the acquired sequence length. As shown in the energy landscape, the sensing window at lengths greater than 54 nt can adapt to differing structures. Low energy barriers exist between each of the structures and isomerization between folds can occur. In addition, alternative structures slow formation of the ligand-competent structure. This change to a “rougher” landscape topology is in alignment with kinetic experiments of the full length riboswitch.

## Discussion

We have developed a computational approach based on energy landscapes that describes riboswitch conformational ensembles at different sequence ranges as the riboswitch elongates to full length and analyzed the resulting landscapes for ten riboswitches. Our analysis suggests variations in riboswitch control of gene regulation and patterns that describe either kinetic or thermodynamic control.

In the case of kinetic control of riboswitches, base-pairing occurs within milliseconds as the RNA emerges from the RNA polymerase and the ligand-competent aptamer structure emerges in the sensing window. This ligand-competent structure is in equilibrium with other non-ligand-competent structures. Exchange between stable states occurs up until the downhill folding window, with either bound or unbound ligand, and the final RNA structure remains stable during the functional window.

In the alternative thermodynamic control, structural changes can be induced by the ligand throughout the elongation process. Other studies by Nudler et al. [19], [68] as well as Edwards et al. [83] have made similar suggestions. Our approach is unique in that it can predict, for multiple riboswitch classes, distinct windows in the folding pathway and related them to gene regulation.

Because all RNA folding algorithms are imperfect, and sampling of suboptimal structures at elevated temperatures entails additional approximations, the results presented here are subject to these standard uncertainties. We also cannot account for all biologically relevant events, such as transcription pause sites [22], [47], and the algorithms we use for RNA pseudoknots (pknotsRG [61]) are limited to simple recursive pseudoknots. Still, the overall trends for many riboswitches appear robust and helpful for interpretation of riboswitch mechanisms. Figure S1 shows significant agreement of our predicted 2D riboswitch structures to 2D structures of known native states. To further explore further our 2D models, we also perform illustrative 3D folding on two riboswitch aptamers (see Text S1, Figure S2).

Support for switching states comes from our energy landscape analysis, where shorter-than-full length riboswitch transcripts form different low-energy structures in the sensing window. The low energy barrier and natural tendency for fast RNA base-pairing implies rapid structural interconversions at ambient temperatures. Indeed, thermal excitation for overcoming an energy barrier of 5–10 kcal/mol has been postulated for the *pbuE* aptamer [30].

Another remarkable property of the sensing window is that the ligand-bound conformation is the minimum energy structure on the energy landscape. As a result of the aptamer's upstream location on the riboswitch construct, the ligand-competent state forms as soon as it emerges from the polymerase. Some studies suggest that ligand-competent states do not occur spontaneously in the majority of riboswitches [27], though our computations suggest that ligand-competent aptamers form in all except two of the riboswitches (*pbuE* and preQ1) examined here. Even for these riboswitches, ligand-competent structures exist within the cluster of accessible conformations though. The centroid of a cluster may be closer to the reference standard RNA structure [84].

We also postulate that the ability of the RNA to rearrange itself is marked by a funnel landscape, as in the downhill folding window, with small energy barriers (<5 kcal/mol). This property exists in other structurally rearranging RNAs, such as the *E. coli* phage MS2 RNA, which requires rearrangement in order to restrict ribosome binding and control gene expression (Figure S3) [85]. The synthetic MDV-1 RNA also rearranges hairpins during transcription and has a downhill folding landscape [86]. In fact, exactly at the length where a new hairpin is favored, a steep funnel landscape forms towards the mfe (Figure S4). These energy landscape properties support a model where fast isomerization to the mfe is possible in a downhill folding landscape.

An essential function of riboswitches is their ability to transmit ligand binding into gene regulation. Kinetically-controlled riboswitches perform binding and structural rearrangement during transcription, while thermodynamically-controlled riboswitches display structural reversibility in response to ligand post transcription [87]. A recent study by Lemay et. al. suggested that the difference between kinetic and thermodynamic control of ligand binding is a byproduct of the mechanism of gene control (transcriptional or translational regulation) [87]. Based on our analysis, both transcription terminator riboswitches and SD-sequestering riboswitches can utilize either mechanism. Furthermore, kinetic or thermodynamic control depends on the sequence length during which the riboswitch is sensitive to the ligand, the sensing window, where both ligand-competent and non-ligand-competent structures co-exist. When the sensing window occurs early, kinetic control is suggested. When the sensing window occurs in the full length riboswitch, thermodynamic control rules. Studies have shown that most full length transcription terminator riboswitches cannot undergo ligand-dependent conformational changes [64]. These findings suggest kinetic control *in vivo*. Our computational results support this and broaden this notion to some translationally-acting riboswitches as well (*thiM* and *moaA*). If the ligand binding regime of the sensing window does not occur at full length, then the riboswitch cannot bind ligand reversibly and undergo the requisite structural modulation.

Designing riboswitches responsive to novel ligands and capable of novel functions has been an area of active research [88]–[93]. The field of RNA design has traditionally relied on generating massive sequence pools (e.g. SELEX) evolved over time to bind a specific target. Computational design methods have been developed to aid this process, implementing nucleotide transition matrices and motif filtering to generate diverse structure pools [94], [95]. For example, Luo et. al. [96] developed RNA pools with high complexity through repeated mutation and filtering exclusively on junction sequences. These methods have now been advanced to combine 2D with 3D folding algorithms in tandem to screen for target structures [97]. Using only folding algorithms, Chushak and Stone were able to confirm the sequences of six aptamers generated by *in vitro* methods. To further explore further our 2D models, we also perform illustrative 3D folding on two riboswitch aptamers (see Text S1). Robust, quantitative methods in riboswitch design are still lacking, and the ability to screen sequences computationally is of particular interest. Our analysis suggests that replicating the order of landscape windows could serve as a guide for tailored riboswitch function. Specific design strategies will be the focus of future work.

## Materials and Methods

### RNA folding and energy landscapes

Prediction of secondary structure from primary sequence is based on a set of thermodynamic parameters, determined from UV absorbance or calorimetric measurements adjusted with temperature [34], [62]. RNA folding predictions can predict the correct structure with 73% accuracy for sequences of less than 300 nucleotides [98], [99]. The goal of secondary structure prediction is to find the minimum free energy (mfe) structure, as well as other low-energy, probable structures. The mfe is thus the structure at highest concentration at equilibrium. The widely used algorithms of Zuker and Stiegler assign free energy increments to each base-pairing or stacking interaction and penalties to constraining conformations [100]. For a comprehensive review of RNA secondary structure prediction see [101].

Here, secondary structure prediction was performed using RNAfold [102] from the Vienna RNA package [60] as well as *mFold* web server [103]. For the prediction of pseudoknots (i.e., nested hydrogen-bonding base pairings), we use pknotsRG [61]. This program utilizes the same energy model as described above, provides complete suboptimal folding states, and efficiently maneuvers the search space through restriction to a canonical set of rules. Specifically, it: (1) requires that both strands of all helices must be of equal length, (2) minimizes intervening single stranded regions between helices, and (3) draws an arbitrary boundary between competing helices that may overlap. The program was initially tested on the *tenA* TPP riboswitch, and it produced similar results as RNAfold [57].

### RNA sequences

Riboswitch sequences were initially found in the experimental publication as listed in Table 1 and later confirmed by genomic sequences extracted from GenBank via BLAST (see Dataset S1 for a complete listing of sequences and accession numbers). We found slight alterations between published and genomic sequences. This was likely due to experimental requirements, e.g., for amenable primer sequences point mutations were introduced into the sequence. For our purposes, we did not risk any point mutations that might bias the energy landscape, so genomic sequences were followed.

### Suboptimal structure sampling and comparison

Suboptimal structure sampling [104] was performed in our previous study [57] at elevated temperatures from the Boltzmann-weighted distribution of secondary structures. This was required to generate diverse structures that reflected the alternative states of the RNA. Since we now broaden our analysis to RNAs of varying lengths, we have developed a standardized method to generate structures at a prescribed set of temperatures. Longer RNAs with greater probability of base-pairing have a higher melting temperature than shorter RNAs [105]. Thus, using a single standard temperature for all RNAs was not possible. To overcome this, we calculate the predicted melting temperature using RNAheat [102], defined as the maximum specific heat on the temperature curve. Using RNAsubopt, we sample 100 structures at physiological temperature (37°C), followed by 150 structures six deciles towards the melting temperature. For example, if the melting temperature is 90°C, we sample 100 structures at 37°C, followed by 150 structures at 43°, 49°, 55°, 61°, 66°, and 72°, respectively, to produce a complete sample of 1,000 structures. This method produces a sample of structures diverse enough to produce the alternative conformations found by experimental studies, but also maintains the accuracy of the mfe and structures at the low-energy portion of the landscape. For pknotsRG, we compute the mfe and used complete reporting of suboptimal structures within 10 kcal/mol of the minimum free energy (pknotsRG -m -s -e 10). Free energy of folding for all structures generated is then re-calibrated at 37°C using RNAeval to be physiologically relevant and for purposes of comparison. To attain a representative view of the energy landscape through time, we iteratively sample and measure the energy landscape from 64% of the full length of RNA, in increments of one nucleotide. Prior to 60% of the full length RNA, we found that most riboswitches are composed solely of the RNA aptamer domain, and thus would not reflect the dual-structure nature of the landscape through time.

Structure comparison was performed using the base pair distance in RNAdistance [57], [60], [104]. The base-pair distance measures the number of base pairings that require breaking or forming in order to convert one structure into another (Figure 1d for example). For pseudoknot structure comparisons, we use the base pair distance as well. Initially, the total number of pseudoknot base pairs is summed and recorded for each structure. The structure is subsequently removed of all pseudoknots, to produce a standard Vienna RNA in dot-bracket notation [60], [102]. The set of structures is then compared using RNAdistance as described above, providing the base pair distance between structures. After base pair distance calculation, pseudoknots are subsequently replaced for all further analysis.

### Energy landscape analysis

Free energy of folding versus base pair distance is used as an approximate measure of the energy landscape. Once the landscape is generated, clustering is performed in the statistical software R [106] using the cluster package. We used Partitioning Around Medioids (PAM), a partition clustering algorithm, specifying the number of clusters to be two (*k = 2*). This method is highly effective and amenable to our methods as it predicts clusters by minimizing a sum of dissimilarities, which we deal with implicitly as the base pair distance. For quantifying the strength of clustering and as a surrogate quantity for the presence of two clusters, the average silhouette width was used with a threshold of ≥0.4. The average silhouette coefficient for all points (SC) ranges from 0 to 1, indicating a poor or well-clustered result respectively. For a full description, see ref [107].

### Energy landscape window definitions

We generate energy landscapes for all riboswitch sequence lengths from 64% of full length until the end of the expression platform. We then analyze the landscape for the presence of clusters and particular shapes. Barrier-limited landscapes are defined by the following: (1) alternative, low-energy structures within 2.0 kcal/mol of each other (2) an energy barrier between 5 and 10 kcal/mol between the two states (3) a funnel-type topology within the vicinity of the local minima. Using the criteria above, a “sensing window” is defined by at least 5 consecutive nucleotide lengths that meet the definition of “barrier-limited.” We also found that, within the same RNA construct, the energy landscape may differ. Those landscapes that did not fit the criteria of barrier-limited landscape and had a barrier of <5 kcal/mol are defined as “funnel” and if the barrier is >10 kcal/mol defined as “cluster.” If five or more consecutive nucleotides display similar funnel landscapes, it is a “downhill folding window.” Conversely, if five or more consecutive nucleotides display cluster landscapes and the functionally-opposing structures occupy the sets, it is a “functional window.

## Supporting Information

Figure S1 **Comparison between computationally predicted and experimentally verified structures.** Comparison between computationally predicted and experimentally verified structures. Ligand binding domains of experimentally validated structures are shown. Nucleotides which differ between the two structures are marked in blue (predicted structure is base-paired), red (predicted structure is single-stranded), orange (predicted structure alternatively base-paired), and green (agreement). Dashed lines represent base pairs present in the computationally predicted structure.
(EPS)

Figure S2 **Predicted tertiary structures of the SAM and** ***thiM*** **TPP aptamer.** Predicted tertiary structures of the SAM and thiM TPP aptamer. (a) Overlap of the predicted and wild-type SAM aptamer of *Thermoanaerobacter tengcongensis* in the ligand-free form. (b) RMSD trajectory (difference between all C3′ atoms in wild-type and predicted) over the course of the 3D folding. (c) Overlap of the ligand-free predicted and ligand-bound wild-type TPP aptamer of *Escherichia coli*. (d) RMSD trajectory (difference between all C3′ atoms in wild-type and predicted) over the course of the 3D folding.
(EPS)

Figure S3 **Folding pathway for the** ***E.coli*** **phage MS2 RNA.** Initially, two structures are possible which the RNA may fold into (86–117 nt). The RNA was found to isomerize into the bottom structure. The energy landscape from 122–136 nt is downhill folding and accommodates such isomerization to this specific mfe structure.
(EPS)

Figure S4 **The folding pathway of the synthetic MDV1 RNA.** The following is an explanation from the original article by Kramer and Mills:Both hairpin B and hairpin T are present during chain growth but cannot coexist on the same molecule. Hairpin B is initially favored during chain growth, prior to formation of hairpin T. Once MDV1 grows sufficiently long (61–71 nt), hairpin T is more stable than hairpin B. Additional chain growth leads to the dissociation of hairpin T in favor of the formation of hairpin C and the reformation of hairpin B, which together contributes more to the stability of the RNA than hairpin T alone. (Kramer et al. Nucleic Acids Research 1981 9:5109–5124.) The energy landscape from 76–96 nt favors downhill folding and thus isomerization to the structure containing hairpin B and hairpin C.
(EPS)

Figure S5 **Clusters in the energy landscape as a function of sequence length.** Charts display the number of clusters in the energy landscape at 1 nt resolution. Significant clusters were defined by a silhouette coefficient ≥0.4 (see Materials and Methods).
(EPS)

Table S1 **Tertiary contacts used as input into NAST.**
(DOC)

Text S1 **Potential 3D structures of aptamers arise as an extension of 2D predictions.**
(DOC)

Dataset S1 **Wild-type riboswitch sequences used in this study.**
(DOC)
