# Supplementary material for: Dynamic Energy Landscapes of Riboswitches Help Interpret Conformational Rearrangements and Function
Source: PLoS Comput Biol. 2012 Feb 16;8(2):e1002368. doi: 10.1371/journal.pcbi.1002368 (PMC3280964; doi:10.1371/journal.pcbi.1002368)
Supplement: Table S1 — Tertiary contacts used as input into NAST. (DOC) [file pcbi.1002368.s006.doc]

| Table S1. Tertiary Interactions Used to Model 3D Structures by NAST. |
| --- |
| Nucleotides in tertiary interaction |
| *thiM TPP Riboswitch Aptamer* |
| G16:A84 |
| G19:A47 |
| U54:U79 |
| G16:C55 |
| A43:U39 |
| A53:A84 |
| A56:G17 |
| T.tengcongensis SAM-I Aptamer |
| A9:A46 |
| A10:A45 |
| A24:U64 |
| C25:G68 |
| U26:U67 |
| G27:C66 |
| G28:C65 |
| A45:A46 |
| A45:U57 |
| A46:U57 |
| U64:A85 |
