# Supplementary material for: Dynamic Energy Landscapes of Riboswitches Help Interpret Conformational Rearrangements and Function
Source: PLoS Comput Biol. 2012 Feb 16;8(2):e1002368. doi: 10.1371/journal.pcbi.1002368 (PMC3280964; doi:10.1371/journal.pcbi.1002368)
Supplement: Text S1 — Potential 3D structures of aptamers arise as an extension of 2D predictions. (DOC) [file pcbi.1002368.s007.doc]

**Supplementary Information**

Giulio Quarta 1,3, Ken Sin 1, Tamar Schlick 1,2^*^

1 Department of Chemistry, New York University, New York, United States of America

2 Courant Institute of Mathematical Sciences, New York University, New York, United States of America

3 Howard Hughes Medical Institute - Medical Research Fellows Program
*Email: schlick@nyu.edu

Text S1 – Potential 3D structures of aptamers arise as an extension of 2D predictions.

Dataset S1 – Wild-type riboswitch sequences used in this study.

Table S1 – Tertiary contacts used as input into NAST.

Figure S1 – Comparison between predicted and experimentally validated 2D structures.

Figure S2 – Predicted tertiary structures of the SAM-I and *thiM* TPP aptamer.

Figure S3 – Structures and energy landscapes of the *Escherichia coli* phage MS2 RNA.

Figure S4 - Structures and energy landscapes of the MDV-1 RNA.

Figure S5 – Energy landscape clustering plotted by length of transcription.

Potential 3D structures of aptamers arise as an extension of 2D predictions.

Conformational changes in the aptamer domain also induce changes in local pairing interactions within the ligand binding pocket. To explore further our 2D models, we performed exploratory 3D folding simulations of two aptamer domains starting from the pre-existing secondary structures. The SAM-I aptamer of *Thermoanaerobacter* tengcongensis, has been crystallized in the ligand-free form (PDB 3IQP) [1] and we thus choose it for 3D folding prediction. We also choose the thiM aptamer domain (PDB 2GDI) for tertiary folding predictions [2].

NAST uses both 2D and 3D contact information to improve the quality of structural predictions [3]. Secondary structure information is taken from the energy landscape described in the manuscript and input into NAST. For tertiary contacts, we use information from SHAPE and in-line probing experiments, as listed in Table S1. Contacts reported in the literature were chosen based on their importance in folding. Since we do not know exact values, distances between atoms involved in the tertiary interaction were set to a default value of 1.3 nm and the spring constant was set to 200 kJ/nm^2^. We choose these values based on the recommendations of NAST developers. With this secondary (Figure S1) and tertiary (see Table S1) information we run NAST for twenty million time steps at a default temperature of 310K. RMSD comparison was performed in VMD [4] after full alignment of the native crystal structure and predicted structure using backbone C3′ atoms.

As shown in Figure S2, the overall trends in the 3D structure of the aptamer domains can be obtained. The RMSDs comparing the computed to crystal structures are not small (average RMSD ~12Å ± 1 for SAM and ~18Å ± 3 for thiM) but RMSDs for RNAs are much larger than for proteins [5,6]. For thiM, TPP intercalates between P2 and P5, bringing the helices together [2]. We attribute the larger structural distance to the tertiary interactions induced upon TPP binding. These two very preliminary folding experiments indicate that 3D modeling has some potential as a tool combined with 2D energy landscape views. Much more work, of course, is required in this modeling aspect.

Reference List

1. Stoddard CD , Montange RK , Hennelly SP , Rambo RP , Sanbonmatsu KY, et al. (2010) Free state conformational sampling of the SAM-I riboswitch aptamer domain. Structure 18: 787-797.

2. Serganov A , Polonskaia A , Phan AT , Breaker RR , Patel DJ (2006) Structural basis for gene regulation by a thiamine pyrophosphate-sensing riboswitch. Nature 441: 1167-1171.

3. Jonikas MA , Radmer RJ , Laederach A , Das R , Pearlman S, et al. (2009) Coarse-grained modeling of large RNA molecules with knowledge-based potentials and structural filters. RNA 15: 189-199.

4. Humphrey W , Dalke A , Schulten K (1996) VMD: visual molecular dynamics. Journal of Molecular Graphics 14: 33-38.

5. Laing C , Schlick T (2011) Computational approaches to RNA structure prediction, analysis, and design. Curr Opin Struct Biol 21: 306-318.

6. Laing C , Schlick T (2010) Computational approaches to 3D modeling of RNA. J Phys Condens Matter 22: 283101.
