# Supplementary material for: Dynamic Energy Landscapes of Riboswitches Help Interpret Conformational Rearrangements and Function
Source: PLoS Comput Biol. 2012 Feb 16;8(2):e1002368. doi: 10.1371/journal.pcbi.1002368 (PMC3280964; doi:10.1371/journal.pcbi.1002368)
Supplement: Dataset S1 — Wild-type riboswitch sequences used in this study. (DOC) [file pcbi.1002368.s008.doc]

**Dataset S1. Wild-type riboswitch sequences used in the study.**

Sequences include aptamer domain and expression platform according to published structures. FASTA format with GenBank accession numbers and organism names.

>mgtE_Mg

>gi|225184640: 1395621-1395833 Bacillus subtilis subsp. Subtilis str. 168 complete genome

ACTTCGTTAGGTGAGGCTCCTGTATGGAGATACGCTGCTGCCCAAAAATGTCCAAAGACGCCAATGGGTCAACAGAAATCATCGACATAAGGTGATTTTTAATGCAGCTGGATGCTTGTCCTATGCCATACAGTGCTAAAGCTCTACGATTGAAGGCGCCCGCACGCTTTTTTTGCCGTGCTTCTTTCACCTTCAATCCCGAAGGCTTTTTTT

>tenA_TPP

>gi|225184640:1242238-1242427 Bacillus subtilis subsp. Subtilis str. 168 complete genome

GCAGAACAATTCAATATGTATTCGTTTAACCACTAGGGGTGTCCTTCATAAGGGCTGAGATAAAAGTGTGACTTTTAGACCCTCATAACTTGAACAGGTTCAGACCTGCGTAGGGAAGTGGAGCGGTATTTGTGTTATTTTACTATGCCAATTCCAAACCACTTTTCCTTGCGGGAAAGTGGTTTTTTTA

>ECOL_Moco_moaA

>gi|294489418: 816533-816664 Escherichia coli IHE3034, complete genome

GGAACCACTAAACACTCTAGCCTCTGCACCTGGGTCAACTGATACGGTGCTTTGGCCGTGACAATGCTCGTAAAGATTGCCACCAGGGCGAAGGAAGAAATGACTTCGCCTCCCGTATCTGGAAAGGTGTACAT

>ECOL_thiM_TPP

>dbj|AP009048.1| Escherichia coli str. K12 substr. W3110 DNA, complete genome

ACGACTCGGGGTGCCCTTCTGCGTGAAGGCTGAGAAATACCCGTATCACCTGATCTGGATAATGCCAGCGTAGGGAAGTCACGGACCACCAGGTCATTGCTTCTTCACGTTATGGCAGGAGCAAACTATGCAAGTCGACCTGCTGGGTTCA

>add_Adenine

>gi|37509038:1130426-1130651 Vibrio vulnificus YJ016 DNA, chromosome II, complete sequence

TTCGGCGATCAACGCTTCATATAATCCTAATGATATGGTTTGGGAGTTTCTACCAAGAGCCTTAAACTCTTGATTATGAAGTCTGTCGCTTTATCCGAAATTTTATAAAGAGAAGACTCATGAATTACTTTGACCTGCCGAAGATCG

>xpt_Guanine

>gi|633168|emb|X83878.1| B.subtilis xpt and pbuX genes

AATATAATAGGAACACTCATATAATCGCGTGGATATGGCACGCAAGTTTCTACCGGGCACCGTAAATGTCCGACTATGGGTGAGCAATGGAACCGCACGTGTACGGTTTTTTGTGATATCAGCATTGCTTGCTCTTTATTTGAGCGGGCAATGCTTTTTTTA

>ydhL_pbuE_Adenine

>gi|1945083|dbj|D88802.1| Bacillus subtilis DNA for phoB-rrnE-groESL region, complete cds

TCACTTGTATAACCTCAATAATATGGTTTGAGGGTGTCTACCAGGAACCGTAAAATCCTGATTACAAAATTTGTTTATGACATTTTTTGTAATCAGGATTTTTTTTA

>GEMM_CDA

>gi|169637063:c1860182-1860056 Candidatus Desulforudis audaxviator MP104C, complete genome

CGAAAGGGCAAACCGGTACGAAAGTCCGGGACGCAAAGCTACGGGTCCTTAAGTTCCATGGGGAATAGGACGGCTGAGCCGCTGGGGTTATTACTTTCGCGGAGCCGCCCTATGGGGCGGTTTTTAT

>BSUBT_metI_SAM

>gi|225184640:1258192-1259613 Bacillus subtilis subsp. Subtilis str. 168 complete genome

TCAGAAAATTGAAATCGATATTTCTTATCGTGAGAGGTGGAGGGACTGGCCCTTAGAAACCTCAGCAACCGGCTTGTTTTGCATTTGCAAAGCGCCAAGGTGCTAAATCCAGCAAGCGTTTTTTATGCTTGGAAGATAAGAAGAAGCGTTAAACCCCTTCTTCTTATGAAGAAGGGGTTTTTA

>FNUC_PREQ1

>gi|20095250:498154-498222 Fusobacterium nucleatum subsp. Nucleatum ATCC 25586, complete genome

AGTAGATGTGCTAGCAAAACCATCTTTAAAAAACTAGACTTGGGGTGCAAGTCCCCTTTTTTATTGCTT

>MS2_RNA (Groenveld et. al. RNA 1995 1:79-88.)

gggTgggaccccTTTcggggTccTgcTcaacTTccTgTcgagcTaaTgccaTTTTTaaTgTcTTTagcgagacgcTaccaTgcTaTcgcTgTaggTagccggaaTTccaTTccTaggaggTTTgaccTgTgcga

>MDV_1_RNA (Kramer et. al. Nucleic Acids Research 1981 9:5109-5124.)

ggggaaccccccTTcgggggTcaccTcgcgcagcgggcTgcgcgaaggggccacgcTgcgaagcagcgTggcggTTcTcgTggTTaccgaaacgcacgaag
